# CDDO-Imidazolide inhibits growth and survival of c-Myc-induced mouse B cell and plasma cell neoplasms

**DOI:** 10.1186/1476-4598-5-22

**Published:** 2006-06-07

**Authors:** Seong-Su Han, Liangping Peng, Seung-Tae Chung, Wendy DuBois, Sung-Ho Maeng, Arthur L Shaffer, Michael B Sporn, Siegfried Janz

**Affiliations:** 1 Laboratory of Genetics, Center for Cancer Research (CCR), National Cancer Institute (NCI), National Institutes of Health (NIH), Bethesda, MD, USA; 2 Laboratory of Cellular Carcinogenesis and Tumor Promotion, CCR, NCI, NIH, Bethesda, MD, USA; 3 Metabolism Branch, CCR, NCI, NIH, Bethesda, MD, USA; 4 Department of Pharmacology, Dartmouth Medical School, Hanover, NH, USA; 5 Markey Cancer Center, Department of Radiation Medicine, University of Kentucky, Lexington, KY 40536, USA

## Abstract

**Background:**

Gene-targeted iMyc^Eμ ^mice that carry a His_6_-tagged mouse *Myc*(c-*myc*)cDNA, *Myc*^His^, just 5' of the immunoglobulin heavy-chain enhancer, Eμ, are prone to B cell and plasma cell neoplasms, such as lymphoblastic B-cell lymphoma (LBL) and plasmacytoma (PCT). Cell lines derived from Myc-induced neoplasms of this sort may provide a good model system for the design and testing of new approaches to prevent and treat MYC-driven B cell and plasma cell neoplasms in human beings. To test this hypothesis, we used the LBL-derived cell line, iMyc^Eμ^-1, and the newly established PCT-derived cell line, iMyc^Eμ^-2, to evaluate the growth inhibitory and death inducing potency of the cancer drug candidate, CDDO-imidazolide (CDDO-Im).

**Methods:**

Morphological features and surface marker expression of iMyc^Eμ^-2 cells were evaluated using cytological methods and FACS, respectively. mRNA expression levels of the inserted *Myc*^His ^and normal *Myc *genes were determined by allele-specific RT-PCR and qPCR. Myc protein was detected by immunoblotting. Cell cycle progression and apoptosis were analyzed by FACS. The expression of 384 "pathway" genes was assessed with the help of Superarray^© ^cDNA macroarrays and verified, in part, by RT-PCR.

**Results:**

Sub-micromolar concentrations of CDDO-Im caused growth arrest and apoptosis in iMyc^Eμ^-1 and iMyc^Eμ^-2 cells. CDDO-Im-dependent growth inhibition and apoptosis were associated in both cell lines with the up-regulation of 30 genes involved in apoptosis, cell cycling, NFκB signaling, and stress and toxicity responses. Strongly induced (≥10 fold) were genes encoding caspase 14, heme oxygenase 1 (Hmox1), flavin-containing monooxygenase 4 (Fmo4), and three members of the cytochrome P450 subfamily 2 of mixed-function oxygenases (Cyp2a4, Cyp2b9, Cyp2c29). CDDO-Im-dependent gene induction coincided with a decrease in Myc protein.

**Conclusion:**

Growth arrest and killing of neoplastic mouse B cells and plasma cells by CDDO-Im, a closely related derivative of the synthetic triterpenoid 2-cyano-3,12-dioxooleana-1,9-dien-28-oic acid, appears to be caused, in part, by drug-induced stress responses and reduction of Myc.

## Background

2-cyano-3,12-dioxooleana-1,9-dien-28-oic acid (CDDO) and closely related derivatives, such as CDDO-imidazolide (CDDO-Im) [1], are novel synthetic triterpenoids that exhibit potent *in vitro *activity against a wide range of human cancers including lung and ovarian carcinoma [2], acute myeloid leukemia [3], cutaneous T-cell lymphoma [3], chronic lymphocytic leukemia (CLL) [4] and multiple myeloma (MM) [5]. CDDO's anti-neoplastic activity involves a complex set of biochemical pathways that can lead, depending on cell type and context, to induction of cell differentiation and apoptosis [3,5-7], inhibition of cell growth and proliferation [2], distortion of redox balance [8], enhancement of TGF-β signaling [9], and suppression of inflammation [10]. The latter can also involve the non-malignant bystander cells of neoplasia, such as macrophages, in which treatment with CDDO results in inhibition of inducible nitric oxide synthase (iNOS) and cyclooxygenase-2 (COX-2) [11,12]. A newly emerging aspect of tumor inhibition by CDDO with implications for tumor invasion and metastasis is the repression of collagenase [10].

Among CDDO's pleiotropic effects on cancer cells, induction of cell death has received the most attention. Killing of cancer cells by CDDO has been associated with down-regulation of c-FLIP (FLICE inhibitory protein), cleavage of Bid (BH3 interacting death domain agonist), activation of caspases 8 and 3, release of mitochondrial cytochrome c, change in PPAR-γ (proximal proliferator-activated receptor gamma) expression, and inhibition of NFκB (nuclear factor-kappa B) [13-17]. CDDO has been shown to activate extrinsic and intrinsic pathways of caspase-dependent apoptosis. One theory postulates that CDDO-induced production of reactive oxygen species is largely responsible for down regulation of c-FLIP, which results in activation of caspase 8 followed by cleavage of Bid and disruption of mitochondria [13-15]. Activation of Bax (Bcl-2 associated X protein) may enhance this response [6,15]. An alternative theory has linked CDDO-induced cell death to a caspase-independent distortion of intracellular Ca^2+ ^homeostasis. According to this view, CDDO causes sustained elevation in cytoplasmic Ca^2+^, which leads, in turn, to activation of Ca^2+^-dependent enzymes including apoptosis-mediating endonucleases [18].

B-lineage neoplasms that predictably develop in transgenic mice, such as the iMyc^Eμ ^[19] and iMyc^Cα ^[20] gene-insertion strains, may be helpful to elucidate the mechanism by which CDDO-Im inhibits human B cell and plasma cell tumors. To evaluate this possibility, we studied the effects of CDDO-Im in two cell lines, designated iMyc^Eμ^-1 and iMyc^Eμ^-2. These lines were derived from a lymphoblastic B-cell lymphoma and plasmacytoma, respectively, that arose in two different iMyc^Eμ ^mice. Strain iMyc^Eμ ^comprises a model of a certain subset of the human *MYC*- and mouse *Myc*-activating t(8;14)(q24;q32) and T(12;15)(*Igh-Myc*) translocations [19]. Here we show that the CDDO-Im-induced killing of the iMyc^Eμ^-1 and-2 cells was associated with both down-regulation of Myc protein and changes in the expression of genes that play important roles in apoptosis, NFκB signaling, and stress and toxicity responses. These results suggested that iMyc^Eμ^-derived cell lines provide a good pre-clinical model system for the ongoing evaluation of the potential utility of CDDO-Im in the prevention and treatment of human B cell and plasma cell tumors.

## Results

### Features of iMyc^Eμ^cells

Gene-targeted iMyc^Eμ ^mice contain a single-copy mouse *Myc*^His ^(c-*myc*) cDNA that has been inserted in opposite transcriptional orientation (5' to 5') just upstream of the mouse immunoglobulin (Ig) heavy-chain intronic enhancer Eμ. The inserted cDNA also encodes a C-terminal His_6 _tag, which is useful to distinguish message and protein encoded by *Myc*^His ^and normal *Myc *[19]. We have recently shown that heterozygous transgenic iMyc^Eμ ^mice that carry one mutated and one normal *Igh *locus are prone to mature B cell and plasma cell neoplasms including IgM^+ ^lymphoblastic B-cell lymphoma (LBL), Bcl-6^+ ^diffuse large B cell lymphoma, and Ig-secreting CD138^+ ^plasmacytoma (PCT) [19]. To study the growth and survival requirements of iMyc^Eμ ^tumor cells *in vitro*, we derived a cell line from a LBL, designated iMyc^Eμ^-1, and a PCT, designated iMyc^Eμ^-2. The features of the iMyc^Eμ^-1 cells, which over-express *Myc*^His^, as expected, have been described in a previous publication [21]. The features of the iMyc^Eμ^-2 cells are described here.

**Figure 1:**
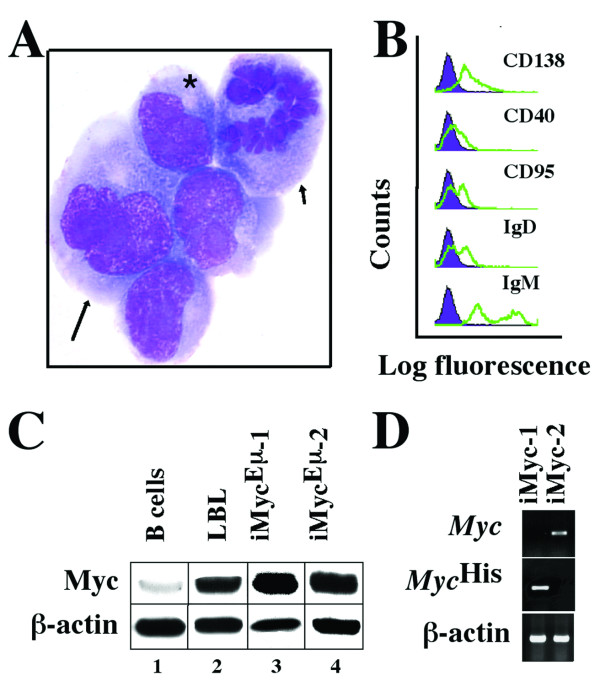
**Features of iMyc^Eμ^-2 cells**. A, Cytofuge specimen stained according to May-Grünwald-Giemsa. A bi-nucleated cell (arrow) and a cell undergoing mitosis (arrowhead) adjoin neoplastic plasmablasts containing the typical paranuclear hof of neoplastic plasmablasts and plasma cells (asterisk). B, B-cell surface marker expression determined by FACS (green lines) compared to isotype controls (purple histograms). C, Western analysis of Myc protein using β-actin as loading control. D, RT-PCR analysis of *Myc *and *Myc*^His ^mRNA compared to β-actin message.

Consistent with their origin from a PCT, the iMyc^Eμ^-2 cells exhibited the typical cytological features of aberrant plasmablasts (Fig. 1A). FACS analysis using a panel of antibodies to cell surface markers (Fig. 1B) showed that the iMyc^Eμ^-2 cells were positive for CD138 (syndecan 1), whereas CD40, CD90 (Fas) and IgD were detectable at lower levels. Expression of IgM showed a biphasic distribution, a feature that is not uncommon among mouse PCT lines that consist of small lymphoid, predominantly diploid cells (IgM^high^) and large plasmacytoid, predominantly tetraploid and often bi-nucleated cells (IgM^low/-^). In agreement with the presence of surface Ig, Southern blotting using JH and Jκ probes revealed rearrangements at the Ig heavy-chain and κ light chain loci (not shown). Western blotting of Myc using antibody that detects both Myc^His ^and normal Myc (Fig. 1C) demonstrated elevated levels of Myc in iMyc^Eμ^-2 cells (lane 4), comparable to the levels in iMyc^Eμ^-1 cells (lane 3) and a randomly chosen primary LBL (lane 2). In contrast, MACS-purified B220^+ ^splenocytes from young, tumor-free iMyc^Eμ ^mice, which were included as control (lane 1), contained low amounts of Myc protein. Molecular cytogenetic studies of the iMyc^Eμ^-2 cells and the primary tumor from which these cells were derived showed that, during the establishment of the cell line, the iMyc^Eμ^-2 cells had changed the mechanism of constitutive Myc expression: in the primary tumor Myc was encoded by the *Myc*^His ^transgene, whereas in the cell line Myc was encoded by the normal *Myc *gene, which was deregulated because of a T(12;15)(*Igh-Myc*) translocation that was not present in the primary tumor [22]. In accordance with this, allele-specific RT-PCR demonstrated that unlike iMyc^Eμ^-1 cells, iMyc^Eμ^-2 cells expressed normal *Myc *(Fig. 1D). These studies established that despite their common origin in the same mouse strain, the iMyc^Eμ^-1 and-2 cells comprise different types of B cells (LBL versus PCT) that rely on different *Myc *genes (*Myc*^His ^versus rearranged, normal *Myc*) to drive cell growth and proliferation.

### CDDO-Im inhibits proliferation and survival of iMyc^Eμ^-1 and-2 cells

**Figure 2:**
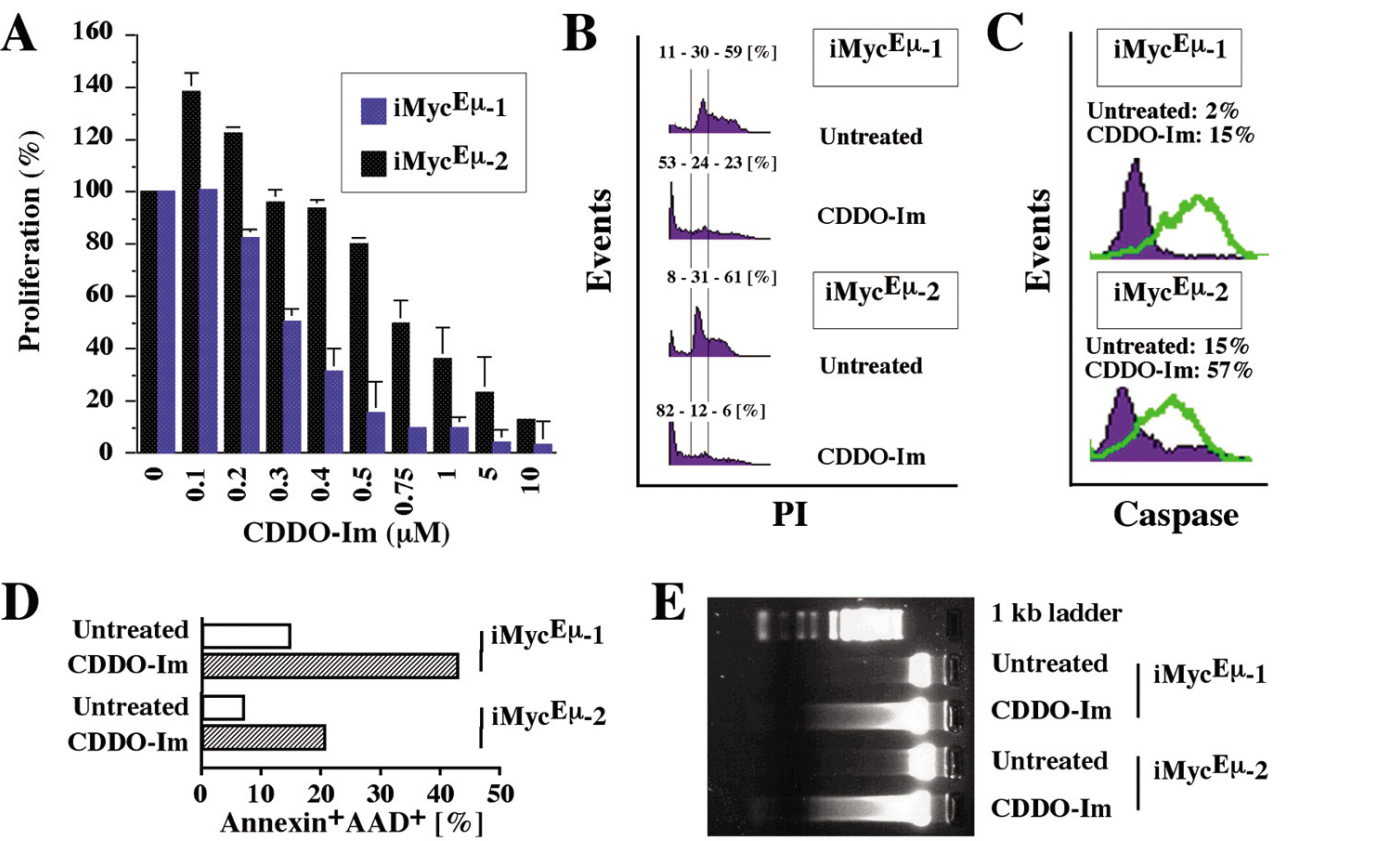
**CDDO-Im-dependent growth arrest and apoptosis in iMyc^Eμ^-1 and-2 cells**. A, MTS assay of cell proliferation, demonstrating growth inhibition in iMyc^Eμ^-1 and-2 cells by ≥200 nM and ≥500 nM CDDO-Im, respectively. Low concentrations of CDDO-Im (100–200 nM) caused growth promotion in iMyc^Eμ^-2 cells by an unknown mechanism. B, Cell cycle arrest and increased number of cells with sub-G0/G1 DNA content, as determined by FACS. The percentage values shown above the histograms indicate the fraction of cells with sub-G0/G1, G0/G1 and S/M DNA content, respectively. C, FACS analysis of cells containing activated caspase 3 upon treatment with CDDO-Im (green lines) or left untreated (purple histograms). D, FACS analysis of CDDO-Im treated cells (open columns) and untreated cells (striped columns) undergoing apoptosis based on annexin V and 7-AAD reactivity. E, Fragmentation of genomic DNA detected by electrophoresis in an agarose gel stained with ethidium bromide.

The anti-proliferative effects of CDDO and CDDO-Im in human cancer cell lines are well documented [2,3,11,16]. To evaluate the potency with which CDDO-Im inhibits the proliferation of mouse iMyc^Eμ^-1 and-2 cells, we treated these cells for 24 hrs with a dose range of CDDO-Im and followed up with the MTS assay. Although CDDO-Im caused a significant reduction in the proliferation of both cell lines, the iMyc^Eμ^-1 cells were more susceptible than the iMyc^Eμ^-2 cells (Fig. 2A). Whereas 500 nM CDDO-Im diminished the growth of iMyc^Eμ^-1 cells by 85%, this concentration was only marginally effective in iMyc^Eμ^-2 cells. In these cells, growth reduction by ~80% required 5 μM CDDO-Im; i.e., approximately ten times the concentration required for an equivalent reduction in the iMyc^Eμ^-1 cells.

Treatment with CDDO-Im resulted in a net decrease in cell numbers (Fig. 2A), providing indirect evidence that the compound induced cell death. To evaluate this further, we determined cell cycle progression of iMyc^Eμ^-1 and-2 cells using flow cytometry. We chose a CDDO-Im dose of 400 nM for the iMyc^Eμ^-1 cells and a dose of 1 μM for the iMyc^Eμ^-2 cells for this and all subsequent experiments. The exposure time of the cells to the compound, which was also kept constant in all subsequent experiments, was 24 hrs. FACS analysis of propidium iodide-stained cells for cellular DNA content (Fig. 2B) showed a decreased proportion of CDDO-Im-treated cells in the S and G2/M phases of the cell cycle (23% in iMyc^Eμ^-1; 6% in iMyc^Eμ^-2) compared to untreated cells (59% in iMyc^Eμ^-1; 61% in iMyc^Eμ^-2). Furthermore, treated samples exhibited an increase in cells with less than diploid DNA content (53% in iMyc^Eμ^-1; 82% in iMyc^Eμ^-2) relative to controls (11% iMyc^Eμ^-1; 8% iMyc^Eμ^-2).

Since an increase in the sub-G0/G1 DNA content is usually associated with apoptosis, we used flow cytometry to determine activated caspase 3 (death executioner, Fig. 2C) and annexin V (indicator of apoptosis-associated cell membrane damage, Fig. 2D) in iMyc^Eμ^-1 and-2 cells. Treatment of iMyc^Eμ^-1 cells with CDDO-Im resulted in a 7.5-fold increase in cleaved caspase-3 reactivity, from 2% to 15%. The increase in iMyc^Eμ^-2 cells was 3.8-fold: 15% versus 57% (Fig. 2C). The corresponding elevation in annexin V reactivity (annexin^+^AAD^+^) was 2.9-fold (44% versus 15%) in the iMyc^Eμ^-1 cells and 3-fold (21% versus 7%) in the iMyc^Eμ^-2 cells (Fig. 2D). The annexin^+^AAD^- ^fraction (incipient apoptosis) and the annexin^-^AAD^+ ^fraction (dead cells) displayed similar CDDO-Im-induced increases (not shown). These results were confirmed by fractionating genomic DNA on ethidium bromide-stained agarose gels, which demonstrated the typical nucleosomal DNA ladder in cells treated with CDDO-Im, but not in untreated cells (Fig. 2E).

### CDDO-Im causes loss of Myc in iMyc^Eμ^-1 and-2 cells

**Figure 3:**
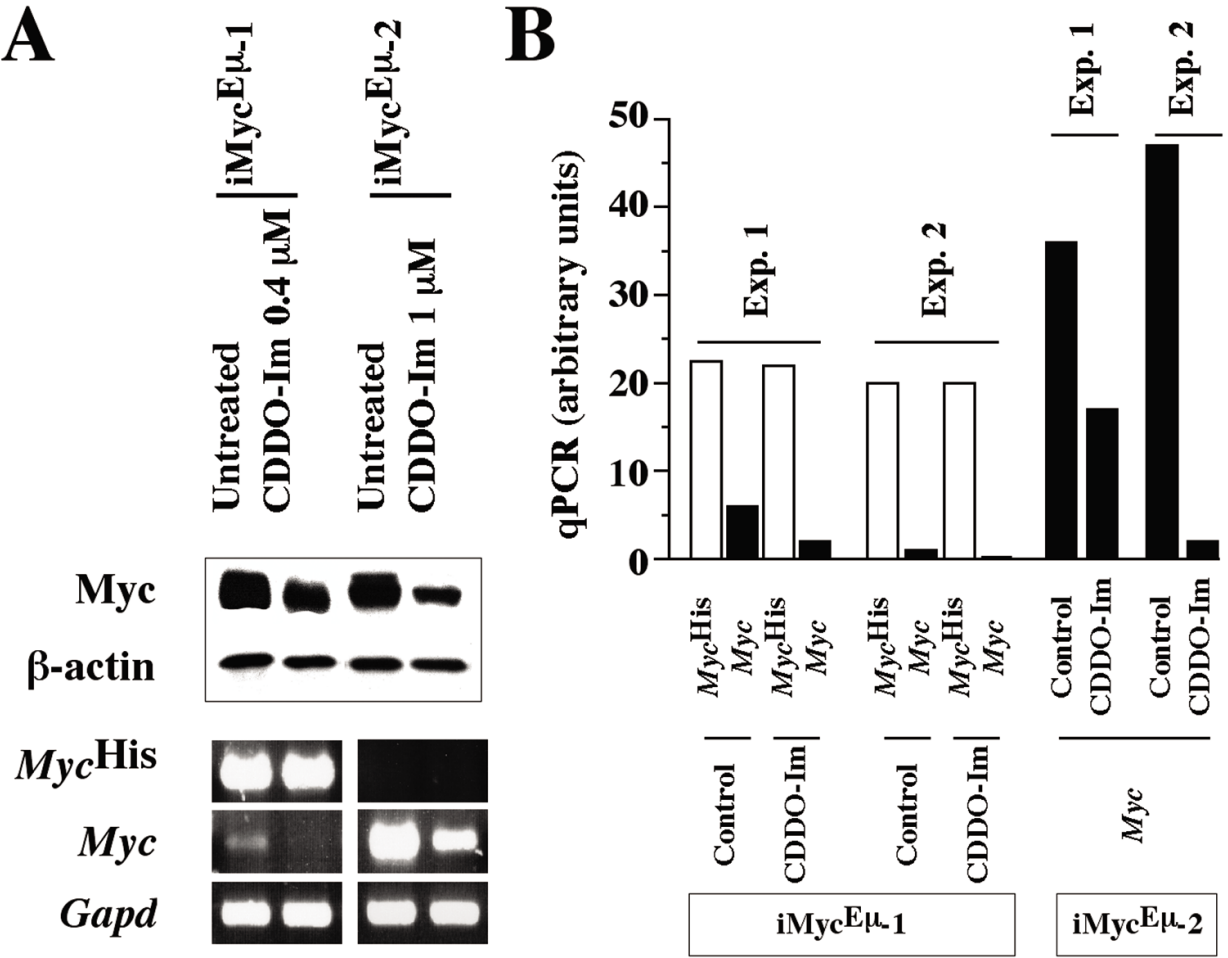
**CDDO-Im-dependent abrogation of NFκB in iMyc^Eμ^-1cells and reduction of Myc in iMyc^Eμ^-1 and-2 cells**. A, Determination of Myc protein using Western analysis (upper panel) and determination of *Myc*^His^, *Myc*, and *Gapd *mRNA using allele-specific RT-PCR (lower panel). B, Measurement of *Myc*^His ^and *Myc *mRNA using allele-specific qPCR.

Because Myc is crucial for growth and proliferation of normal and malignant B cells in humans and mice [23], we evaluated whether Myc expression in iMyc^Eμ ^tumor cells might be negatively affected by CDDO-Im. We treated both cell lines with the compound followed by preparation of cell lysates and Western blotting for Myc, using antibody that detects both Myc^His ^and normal Myc (Fig. 3A top). Comparison of Myc and β-actin, which was included to ascertain equal protein loading, showed that CDDO-Im reduced Myc expression in both cell lines; by a factor of ~3 in iMyc^Eμ^-1 and ~5 in iMyc^Eμ^-2. To determine whether down regulation of Myc protein was associated with a drop in *Myc *message, we performed allele-specific RT-PCR of *Myc*^His ^and *Myc *mRNA. Treatment with CDDO-Im resulted in a reduction of *Myc *transcripts in iMyc^Eμ^-2 cells but did not affect *Myc*^His ^levels in iMyc^Eμ^-1 cells (Fig. 3A bottom). In contrast, the small amount of *Myc *mRNA present in iMyc^Eμ^-1 cells was abrogated by CDDO-Im.

These results were further validated by two independent qPCR analyses that used allele-specific primers to distinguish *Myc*^His ^and *Myc *in the case of the iMyc^Eμ^-1 cells (Fig. 3B left), but only *Myc *primers in the case of the iMyc^Eμ^-1 cells (Fig. 3B right). Consistent with the results in Fig. 3A, the *Myc*^His ^levels (white columns) in the iMyc^Eμ^-1 cells were largely unchanged upon treatment with CDDO-Im, whereas the *Myc *levels (black columns) exhibited a significant reduction. Furthermore, the *Myc *levels in the iMyc^Eμ^-2 cells dropped sharply after exposure to CDDO-Im. These findings indicated that CDDO-Im suppresses Myc protein levels by a post-transcriptional mechanism in the iMyc^Eμ^-1 cells, but a more complicated mechanism that involves transcriptional and post-transcriptional changes in the iMyc^Eμ^-2 cells. Regardless of the precise molecular mechanisms, the reduction in Myc may be an important principle by which CDDO-Im inhibits iMyc^Eμ ^tumor cells.

### CDDO-Im upregulates 30 genes in iMyc^Eμ^-1 and-2 cells

cDNA macroarrays on nylon filter membranes provide a useful screening tool to evaluate the expression of selected pathway genes in mouse cancer cells. To that end, we prepared RNA from iMyc^Eμ^-1 and-2 cells that were either treated with CDDO-Im or left untreated (control). RNA samples were reverse transcribed in the presence of ^32^P-dUTP and hybridized to the gene arrays. This was followed by the determination of the individual gene expression levels and the effects of treatment with CDDO-Im. All samples were evaluated on four different arrays, each containing 96 genes involved in cell cycle regulation, apoptosis, stress and toxicity responses, and NFκB signaling. These arrays were selected because previous work indicated that CDDO-Im can cause growth inhibition (cell cycle array) [2,11,16], cell killing (apoptosis array) [3,4,6,7,13-18], anti-inflammatory effects (NFκB array) [11,12,24] and redox imbalance (stress and toxicity array) [8].

**Figure 4:**
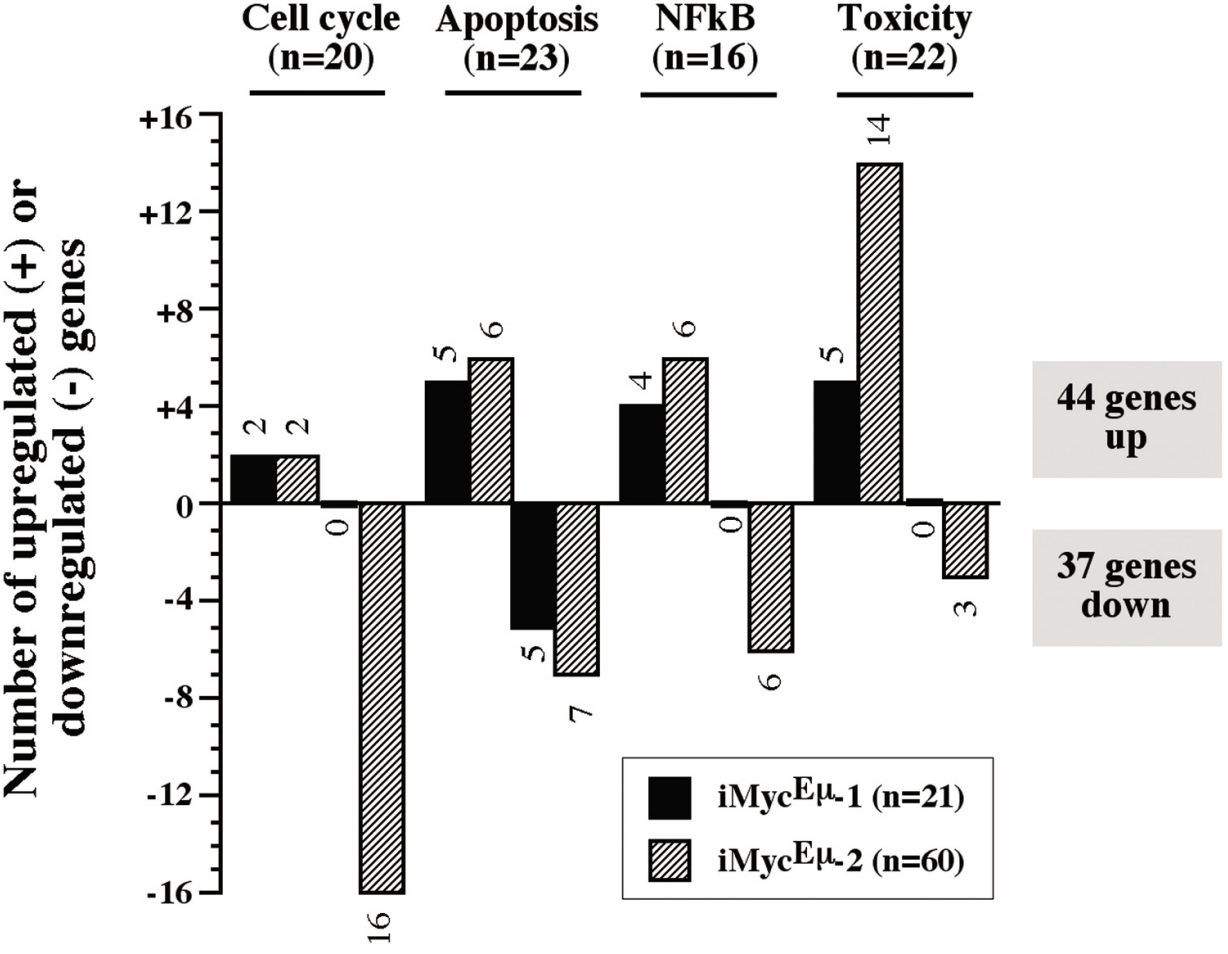
**Number of differentially expressed genes in iMyc^Eμ^-1 and-2 cells upon treatment with CDDO-Im**. Plotted is the overall result of the gene expression analysis using the four cDNA arrays presented in Figure 5. See Additional File 1 for details.

Using stringent criteria for array analysis (≥2-fold expression change reproduced on three or more arrays), we identified a total of 81 differentially expressed genes that were discordantly regulated in CDDO-Im-treated iMyc^Eμ^-1 and-2 cells. The genes were either up or down in either one of the cell lines, but unchanged in the other line (Additional File 1). The distribution of these genes among the four arrays is depicted in Fig. 4. In iMyc^Eμ^-1 cells, 16 genes were up and 5 genes were down. In iMyc^Eμ^-2 cells, 28 genes were up and 32 genes were down. Thus, the iMyc^Eμ^-2 cells responded to CDDO-Im treatment with approximately three times as many changes (60 genes) than the iMyc^Eμ^-1 cells did (21 genes). The distribution of the differentially expressed genes among the four arrays was even: 20 (25%) in the cell cycle array, 23 (28%) in the apoptosis array, 16 (20%) in the NFκB array, and 22 (27%) in the stress and toxicity array. Up-regulated genes (44/81, 54%) slightly outnumbered down-regulated genes (37/81, 46%). In agreement with the RT-PCR and qPCR data presented in Fig. 3, *Myc *was suppressed by CDDO-Im on the cDNA arrays in the iMyc^Eμ^-2 but not the iMyc^Eμ^-1 cells.

**Figure 5:**
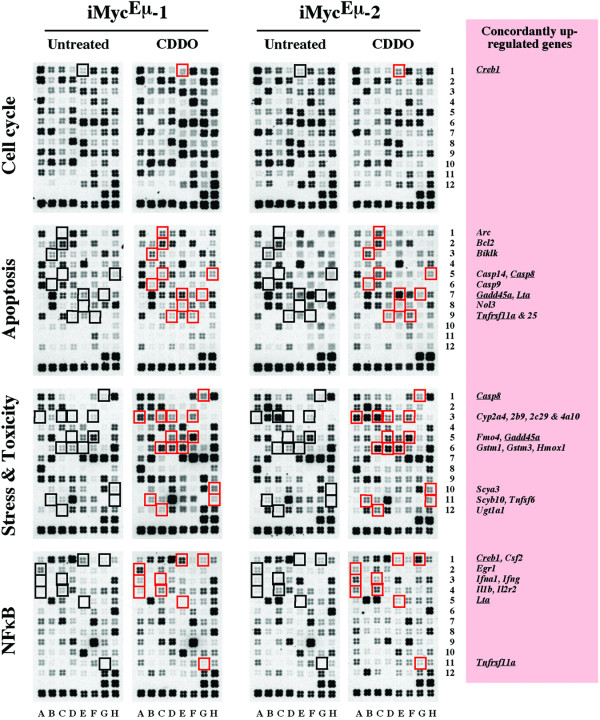
**CDDO-Im-induced up-regulation of genes in both iMyc^Eμ^-1 and-2 cells**. Shown are cDNA arrays containing 96 genes involved in cell cycling (top row), apoptosis (2^nd ^row), stress and toxicity responses (3^rd ^row) and NFκB signaling (bottom row). CDDO-Im-treated and untreated samples are presented as pairs. Indicated by red squares are CDDO-Im-induced genes. The corresponding controls are indicated by black squares to the left. Gene designations are given in the pink text box on the right. Underlined genes were confirmed on two different arrays. Compare Table 1 for additional details.

**Figure 6:**
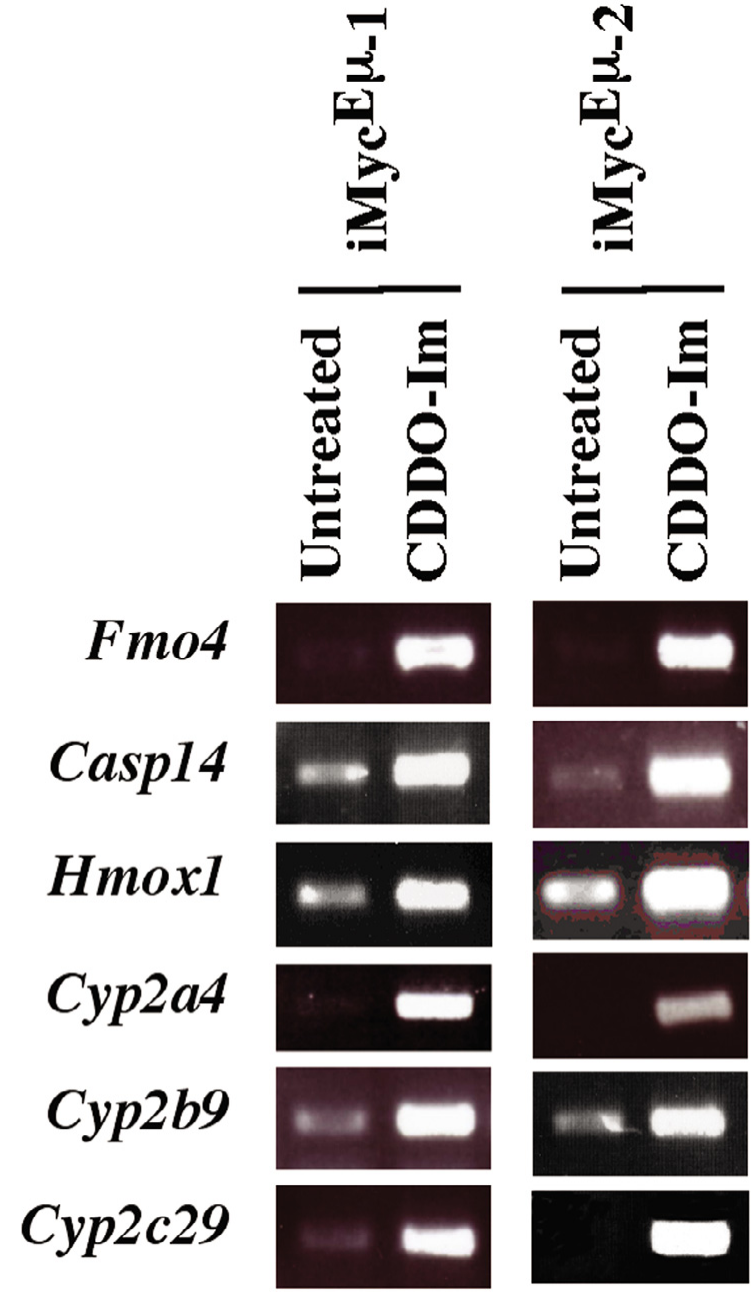
**Verification of gene array results using RT-PCR**. Shown are ethidium bromide-stained PCR fragments of six different genes found to be up-regulated 10-fold or more in CDDO-Im-treated iMyc^Eμ^-1 and-2 cells. The iMyc^Eμ^-1 and-2 cells had been treated for 24 hrs with 0.4 μM and 1 μM CDDO-Im, respectively. The fragments were compared to those detected in untreated cells using the same assay conditions. See Table 1 for further details and Additional File 2 for PCR primers and reaction conditions.

CDDO-Im-induced gene expression changes that occurred concordantly in both cell lines were limited to 30 genes. Interestingly, all of these were up-regulated. They are listed in Table 1 and their location on the array membranes is indicated in Figure 5. Five of 30 genes (underlined in Fig. 5 right) were present on two different arrays, adding confidence to the analysis: *Casp8*, *Creb1*, *Gadd45a*, *Lta *and *Tnfrsf11a*. Six of 30 genes exhibited a 10-fold or higher increase upon exposure of cells to CDDO-Im. Three of these belonged to the subfamily 2 of cytochrome P450 mixed function oxygenases (*Cyp2a5*, *Cyp2b9*, *Cyp2c29*) and the other three encoded flavin-containing monooxygenase 4 (*Fmo4*), heme oxygenase 1 (*Hmox1*) and caspase 14 (*Casp14*), respectively. To verify these observations with an independent method, we performed RT-PCR using the primer pairs and reaction conditions listed in Additional File 2. The CDDO-Im-dependent elevation of all six genes was readily confirmed (Fig. 6).

**Table 1:** Concordantly up-regulated genes upon treatment with CDDO-Im

Gene symbol	Gene name	Gene function	Cell line		Array^1^	Pos.^2^
					
			iMyc-1	iMyc-2		
Arc	activity regulated cytoskeletal-associated protein	CARD family	2.4	47	Apo	C1
Bcl2	B-cell leukemia/lymphoma 2	Bcl2 family	2.2	2.9	Apo	C2
Biklk	Bcl2-interacting killer-like (Bik)	Bcl2 family	2.4	2.0	Apo	B3
Casp8	caspase 8	caspase family	5.5	4.6	Apo	H5
					Tox	G1
Casp9	caspase 9	caspase family	10	6.5	Apo	B6
Casp14	caspase 14	caspase family	15	23	Apo	C5
Creb1	cAMP responsive element binding protein 1	transcription factor	2.1	2.8	Cycle	E1
					NFκB	E1
Csf2	colony stimulating factor 2	cytokine	3.4	5.9	NFκB	G1
Cyp2a4	cytochrome P450, family 2, subfamily a, polypeptide 5	oxidative/metabolic stress	25	20	Tox	A3
Cyp2b9	cytochrome P450, family 2, subfamily b, polypeptide 9	oxidative/metabolic stress	14	14	Tox	C3
Cyp2c29	cytochrome P450, family 2, subfamily c, polypeptide 29	oxidative/metabolic stress	14	434	Tox	D3
Cyp4a10	cytochrome P450, family 4, subfamily a, polypeptide 10	oxidative/metabolic stress	4.9	4.5	Tox	F3
Egr1	early growth response 1	oxidative/metabolic stress	2.5	3.9	NFκB	A2
Fmo4	flavin containing monooxygenase 4	oxidative/metabolic stress	286	145	Tox	D5
Gadd45a	growth arrest and DNA-damage-inducible 45 alpha	ATM/p53 pathway	2.4	2	Apo	E7
					Tox	F5
Gstm1	glutathione S-transferase, mu 1	oxidative/metabolic stress	4.4	16	Tox	C6
Gstm3	glutathione S-transferase, mu 3	oxidative/metabolic stress	2.4	3.3	Tox	D6
Hmox1	heme oxygenase (decycling) 1	oxidative/metabolic stress	10	12	Tox	E6
Ifna1	interferon alpha family, gene 1	cytokine	4.9	7.9	NFκB	A3
Ifng	interferon gamma	inflammation	2.0	2.8	NFκB	C3
Il1b	interleukin 1 beta	inflammation	4.0	11	NFκB	A4
Il1r2	interleukin 1 receptor, type II	inflammation	2.1	2.0	NFκB	C4
Lta	lymphotoxin A	TNF ligand family	4.1	65	Apo	G7
					NFκB	E5
Nol3	nucleolar protein 3 (apoptosis repressor with CARD domain)	apoptosis/necrosis	64	8.4	Apo	E8
Scya3	chemokine (C-C motif) ligand 3 (Mip-1 alpha)	inflammation	5.5	25	Tox	H10
Scyb10	chemokine (C-X-C motif) ligand 10	inflammation	2.7	4.3	Tox	B11
Tnfrsf11a	tumor necrosis factor receptor superfamily, member 11a (RANK)	TNF ligand family	2.7	102	Apo	D9
					NFκB	G11
Tnfrsf25	tumor necrosis factor (ligand) superfamily, member 6 (CD178, CD95L, Fasl, gld)	apoptosis/necrosis	9.6	37	Apo	F9
Tnfsf6	tumor necrosis factor (ligand) superfamily, member 6	TNF ligand family	12	8.2	Tox	H11
Ugt1a1	UDP-glucuronosyltransferase 1 family, member 1	DNA damage/repair	4.6	6.8	Tox	C12

^1^GEArray Q series mouse cDNA gene arrays (SuperArray Bioscience Corporation, Gaithersburg, MD) included the MM-001 cell cycle array (Cycle), MM-002 apoptosis array (Apo), MM-012 stress and toxicity array (Tox) and MM-016 NFκB signaling array (NFκB).

^2^Array position, as indicated in Figure 3

### cDNA microarray analysis reveals additional gene expression changes

The Mouse Lymphochip, a microarray of hematopoietic mouse cDNA clones, provides a tool for extending the above findings at the level of global gene expression [25]. To evaluate the CDDO-Im-dependent changes in the iMyc^Eμ ^cell lines, RNA was obtained from treated and untreated cells, labeled with Cy5-dUTP, and hybridized to the cDNA microarray. An RNA control pool labeled with Cy3-dUTP was co-hybridized to the same array and used as a common denominator by which all samples were compared to one another. Further information on microarray make-up and data interpretation is available on-line [26].

The analysis of three independent RNA samples of iMyc^Eμ^-1 and-2 cells revealed two additional genes (not present on the Superarrays) that were concordantly up-regulated upon treatment with CDDO-Im:*Hck *(hemopoietic cell kinase) and *Spp1 *(secreted phosphoprotein 1). It further uncovered five genes that were concordantly down regulated: *Akt2 *(thymoma viral proto-oncogene 2), *Bat2 *(HLA-B associated transcript 2), *Pim1 *(proviral integration site 1), *Psmb9 *(proteosome subunit, beta type 9) and *Sdc1 *(syndecan 1). One of these genes (*Akt2*) was included (and also found to be reduced) on the Superarrays, but it failed to reach the confidence criteria for significant change. While a more detailed analysis of the microarray results will be presented in a future publication, these results further strengthened the contention that CDDO-Im causes numerous gene expression changes, up and down, in the iMyc^Eμ^-1 and-2 cells.

### CDDO-Im decelerates pristane-induced plasmacytomas in iMyc^Eμ ^mice

**Figure 7:**
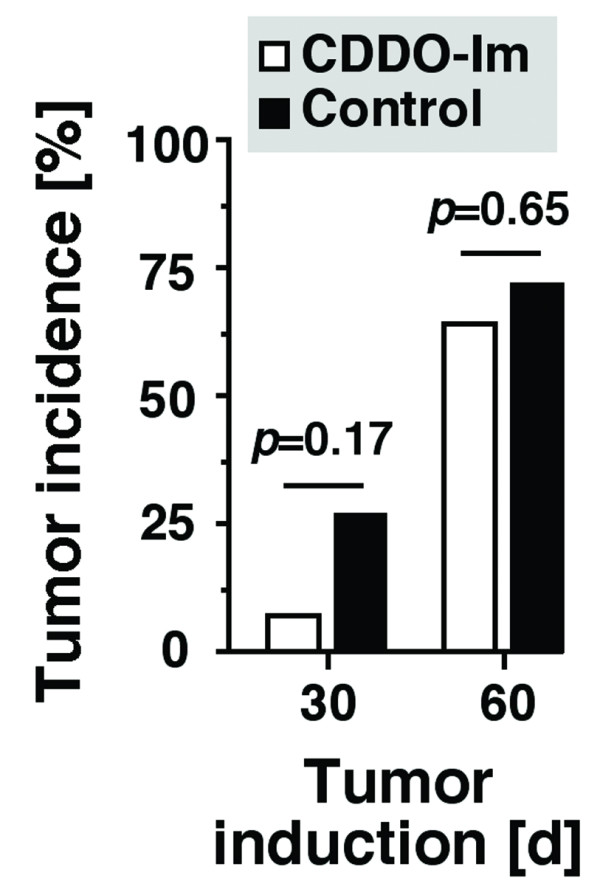
**CDDO-Im inhibits peritoneal plasmacytomas in iMyc^Eμ ^gene-insertion mice**. Mice were injected i.p. on day 1 with 0.2 ml pristane and either treated with CDDO-Im (100 μg per 50-μl i.p. injection, n = 14) or vehicle control (50 μl PEG 400, n = 11). Treatment commenced on day 7 and continued three times per week throughout the observation period (60 days). The diagnosis of plasmacytoma was established on days 30 and 60 post pristane, using stained ascites cell specimens. Tumor incidence was compared using χ^2 ^analysis, the results of which (probability values, *p*) are indicated above the columns.

The growth inhibiting effects of CDDO-Im in the cell lines indicated that this compound might also inhibit *de novo *development of *Myc*-driven B cell and plasma cell tumors *in vivo*. To evaluate this, we primed congenic BALB/c.iMyc^Eμ ^mice with a single i.p. injection of 0.2 ml pristane to undergo inflammation-dependent plasmacytomagenesis in the peritoneal cavity [27]. While only 1 of 14 mice (7.14%) treated with CDDO-Im developed PCT by day 30 of tumor induction, 3 of 11 mice (27.3%) treated with the vehicle control harbored these tumors (Fig. 7 left). A small CDDO-Im-dependent reduction in tumor incidence was also observed on day 60 post tumor induction: 9/14 (64.3%) mice in the CDDO-Im group versus 8/11 (72.3%) mice in the control group (Fig. 7 right). Although none of these differences was statistically significant, owing in large part to the small study groups in this pilot experiment, the apparent deceleration of PCT on day 30 post-pristane suggested that CDDO-Im inhibits tumor development *in vivo*.

## Discussion

This study has demonstrated that CDDO-Im causes growth arrest and apoptosis in the *Myc*-induced mouse B lymphoma and plasmacytoma cell lines, iMyc^Eμ^-1 and iMyc^Eμ^-2. As reported by other investigators for human cancer cell lines, cell killing by CDDO-Im, which is generally more potent than the parental compound CDDO [12], was fast (within 24 h) and efficient (at sub-micromolar concentrations). CDDO-Im-induced apoptosis coincided with a decrease in Myc protein levels and a striking induction of the transcription of genes that are involved in drug metabolism and oxidative stress responses. These genes included *Hmox1 *(heme oxygenase 1), *Fmo4 *(flavin-containing monooxygenase 4) and three representatives of the cytochrome P450 subfamily 2 of mixed-function oxygenases: *Cyp2a4*, *Cyp2b9 *and *Cyp2c29*.

The 10 to 12-fold elevation of *Hmox1 *in CDDO-Im-treated iMyc^Eμ ^cells was consistent with previous reports on the induction of this mouse gene under conditions of chronic [28] or acute oxidative stress [29] and the up-regulation of human HMOX1 by oxidative stress [30,31]. Our finding was also in agreement with a recent study showing that CDDO-Im induces oxidative stress in the neoplastic plasma cells that comprise human MM [8]. In MM, CDDO-Im treatment led to the depletion of glutathione, increased production of reactive oxygen species, and reduction in mitochondrial membrane potential, all indications of heightened oxidative stress. Co-treatment of MM cells with reducing agents, such as N-acetyl-L-cysteine and catalase, alleviated CDDO-Im-dependent oxidative stress, coincident with the elevation of FLICE (Fas-associated death domain-like interleukin-1-converting enzyme) and inhibition of caspase-8 activation [8]. Additional study is warranted to decide whether CDDO-Im-mediated killing of mouse iMyc^Eμ ^tumor cells relies on a mechanism similar to that operating in human MM.

Unlike induction of cytochrome P450 genes, which is common in drug-exposed cells, the more than 100-fold elevation in *Fmo4 *message in CDDO-Im-treated iMyc^Eμ ^cells was surprising, because the flavin-containing monooxygenase family of genes in humans, including *FMO4*, is widely believed to be un-inducible by drugs. Flavin-containing monooxygenase (FMO) proteins are NADPH-dependent microsomal enzymes that catalyze the oxygenation of compounds containing nucleophilic heteroatoms (N-, S-, P- and others) using two-electron transfer chemistry [32]. The broad substrate specificity of FMOs, whose involvement in the metabolism of xenobiotics is increasingly recognized [33], suggests that CDDO-Im might be a substrate of the FMO pathway. More work is required to elucidate the molecular mechanism by which *Fmo4 *is over-expressed in CDDO-Im-treated iMyc^Eμ ^tumor cells and by which Fmo4 might contribute to the catabolism of this compound.

CDDO-Im-treated iMyc^Eμ ^cells contained elevated levels of mRNA encoding caspase 14, an obscure member of the caspase family of proteins. *Casp14 *was up-regulated 15-fold in iMyc^Eμ^-1 cells and 23-fold in iMyc^Eμ^-2 cells. Little is known about circumstances that lead to increased *Casp14 *expression in mice. Consistent with a possible tumor-suppressing function in skin, *Casp14 *was recently shown to be down-regulated in UV-induced skin carcinogenesis [34]. In humans, caspase 14 is mainly found in epidermal cells, in which it can be induced by epigallocatechin-3-gallate, the most abundant tumor-preventive polyphenol in green tea [35]. Unlike human caspase 14, which is a cytokine activator, mouse caspase 14 is a regulator of apoptosis, resembling in enzyme activity and substrate preference apical apoptotic caspases, such as caspase 8 [36]. Considering that activation of caspase 8 has been associated with CDDO-induced killing in human tumor cells [13-15,37], it is conceivable that caspase 14 plays a similar role in apoptosis induction in mouse iMyc^Eμ ^cells. However, this has not yet been shown.

The drop in Myc protein in both iMyc^Eμ ^cell lines suggested that CDDO-Im-induced growth inhibition and apoptosis is regulated, in part, at the level of Myc turnover. Stabilization of Myc has been shown to be key in the growth and survival of Myc-induced B-cell neoplasms in Eμ-Myc mice [38,39], a widely used transgenic model system of the human endemic Burkitt lymphoma t(8;14)/mouse plasmacytoma T(12;15) translocation [40]. The precise mechanism by which CDDO-Im down-regulates Myc in iMyc^Eμ ^cells remains to be elucidated. Given the complexity of Myc protein regulation, this mechanism may involve changes in the PI3K/Akt signaling cascade, specifically in the mammalian target of rapamycin (mTOR) and glycogen-3-synthase beta (G3K-3β) [41-45] components of this cascade. Changes in pathways that include the S-phase kinase-associated protein 2 (Skp2) [46,47] and F-box and WD-40 domain protein 7 (Fbxw7) [48] may also play a role.

Considering Myc's dual ability to activate and repress the transcription of countless target genes [23,49], it is possible that the CDDO-Im-induced loss of Myc is responsible, at least in part, for the observed gene expression changes in the two iMyc^Eμ ^cell lines. According to this hypothesis, the reduction of Myc would relieve the Myc-dependent repression of negatively regulated targets (resulting in their up-regulation) and diminish the activation of positively regulated targets (causing their down-regulation). Of 32 concordantly up-regulated genes in CDDO-Im-treated iMyc^Eμ ^cells (30 on the Superarray + 2 on the Lymphochip), 9 (28%) genes are known Myc targets: *Creb1*, *Bcl2*, *Casp8*, *Casp9*, *Gadd45a*, *Hmox1 *and *Ugt1a1 *[50]. Likewise, one of the five (20%) concordantly down-regulated genes detected on the Lymphochip is a validated Myc target: *Akt2*. Additional studies are required to distinguish a simple association of gene expression change and drop in Myc from a cause-and-effect relationship of Myc levels and gene expression.

## Conclusion

CDDO-Im, the C_28 _imidazolide derivative of CDDO, inhibits tumor cells by a complex mechanism that may rely, in part, on induction of stress responses and down-regulation of Myc. Due to the elusiveness of the cellular targets of CDDO-Im, or its metabolites, the precise molecular mechanism by which the compound affects tumor cells has not yet been elucidated. CDDO-Im is thus at present a promising yet orphan drug candidate for cancer treatment and prevention [1]. A recent study on the collaboration of CDDO-Im with the proteasome inhibitor PS-341 (bortezomib) in apoptosis induction in neoplastic plasma cells has underscored the potential clinical utility of CDDO-Im [5]. The present paper suggests that transgenic mouse models of plasma cell neoplasia, such as the peritoneal PCT that can be readily induced in BALB/c.iMyc^Eμ ^mice [27], may be helpful to further define the mechanism by which CDDO-Im inhibits plasma cell tumors in human beings.

## Methods

### CDDO-Im

CDDO-Im was synthesized by Dr. Tadashi Honda (Dartmouth College, Hanover, NH) [51] and provided by Dr. Edward Sausville (Developmental Therapeutics Program, NCI, Bethesda, MD) through the Rapid Access to Intervention Development (RAID) Program. For biological experiments with iMyc^Eμ^-1 and-2 cells, the compound was dissolved in DMSO and added to cells *in vitro *such that the final DMSO concentration did not exceed 0.1% v/v.

### Derivation and characterization of iMyc^Eμ^-1 and-2 cells

The features and origin of iMyc^Eμ^-1 cells have been described elsewhere [21]. The iMyc^Eμ^-2 cells were derived from a spontaneous plasmacytoma that arose in an iMyc^Eμ ^mouse on the mixed genetic background of segregating C57BL/6 and 129/SvJ alleles. Strain iMyc^Eμ ^develops a high incidence of B cell and plasma cell tumors of different histological types, with plasmacytomas being relatively rare (~20% of tumors). Tissue samples obtained at autopsy were processed for histopathology, which established the diagnosis of plasmacytoma using criteria described in the Bethesda classification of mouse B-cell lineage lymphomas [52]. The iMyc^Eμ^-2 cells were maintained *in vitro *at 37°C and 5% carbon dioxide in RPMI 1640 cell culture medium supplemented with 10% fetal calf serum, 200 mM L-glutamine and 50 μM 2-mercaptoethanol (Gibco-BRL, Rockville, MD). For cytological analysis, cytofuge specimens were stained according to May-Grünwald-Giemsa and inspected by microscopy. For flow cytometry, single-cell suspensions were stained and analyzed on a FACSort^® ^using the CELLQuest™ software (BD Pharmingen, San Diego, CA). Rat anti-mouse CD16/CD32 was used to block FcγII and FcγIII receptors. Antibodies to mouse CD138 (catalog number 553712), CD40 (553787), Fas (CD90, 554255), IgD (553438) and IgM (53519) were purchased from BD Biosciences.

### Western blotting of Myc

Whole cell lysates were obtained by re-suspending pellets of 10^7 ^cells at 4°C for 30 min in RIPA buffer (1% NP-40, 0.5% sodium deoxycholate, 0.1% SDS, 10 ng/ml PMSF, 0.03% aprotinin, 1 μM sodium orthovanadate). The lysates were centrifuged for 6 min at 14,000 g and the supernatants were stored at-70°C as whole cell extract. Protein concentrations of extracts were determined using the BCA kit (Bio-Rad, Richmond, CA). For Western blotting, 40 μg of extract was resolved electrophoretically in denaturing 10% SDS-PAGE gels and transferred by electroblotting to nitrocellulose membranes. Membranes were probed with antibody to Myc (sc-764) from Santa Cruz Biotechnology (Santa Cruz, CA) using titers from 1:1000 to 1:5000. The positions of the proteins were visualized with horseradish peroxidase-conjugated secondary antibody (Amersham, 1:5000) using the chemiluminescence detection kit from Amersham. To confirm equal loading, the membranes were stripped and re-probed using an antibody specific for β-actin (sc-8432, Santa Cruz Biotechnology).

### Allele-specific RT-PCR of Myc and Myc^His ^mRNA

For semi-quantitative determination of *Myc *and *Myc*^His ^mRNA, total RNA was isolated using TRIzol (Sigma, St. Louis, MO, USA). The integrity of RNA was verified by electrophoresis. Double stranded cDNA was synthesized from 1 μg of total RNA, using the AMV Reverse Transcriptase kit (Roche, Indianapolis, IN). A common 5' primer for both *Myc*^His ^and *Myc *(5'-TCT CCA CTC ACC AGC ACA AC-3') was combined with a specific 3' primer for *Myc*^His ^(5'-CCT CGA GTT AGG TCA GTT TA-3') and *Myc *(5'-ATG GTG ATG GTG ATG ATG AC-3') to distinguish the two messages. Thermal cycling conditions were as follows: 95°C for 5 min (initial template denaturation) followed by 20 cycles of amplification at 57°C (primer annealing), 72°C (extension) and 95°C (melting), each for 1 min. PCR amplification of *Aktb *cDNA was performed for each sample as a control using the following primer pair: 5'-GCA TTG TTA CCA ACT GGG AC-3' (forward) and 5'-AGG CAG CTC ATA GCT CTT CT-3' (reverse). PCR products were analyzed by electrophoresis in 1% agarose gel and visualized by staining with ethidium bromide.

In some experiments, *Myc *and *Myc*^His ^mRNA were determined using real-time, quantitative RT-PCR (qPCR) using the SYBR Green I method on the Light Cycler (Roche) with attendant software for analyzing fluorescence emission data. The reaction volume (20 μl) contained 100 ng cDNA, the Light Cycler Fast Starter mix, 1 mM MgCl_2 _and primers. Thermal cycling conditions were as follows: 95°C for 10 min (initial template denaturation) followed by 40 cycles of amplification at 57°C (primer annealing), 72°C (extension) and 95°C (melting), each for 1 min. PCR amplification of *Gapd *cDNA was performed as control.

### Proliferation and cell cycle analysis

Proliferation was determined with the help of the "MTS" Cell Titer 96 aqueous non-radioactive cell proliferation assay from Promega (Madison, WI) following the manufacturer's protocol. Briefly, 3 × 10 iMyc^Eμ ^1 or-2 cells were re-suspended in 100 μl RPMI 1640 supplemented with 10% FBS, 100 U/ml penicillin, 100 μg/ml streptomycin and 25 μg/ml LPS and placed into 96-well flat-bottom microtiter plates (Costar, Cambridge). After incubation for 20 hrs at 37°C and 5% CO_2_, 20 μl of MTS/PMS solution was added to each well. The cells were incubated for another 4 hrs and the absorbance at 490 nm was measured using an ELISA reader. For cell cycle analysis, cells were stained with 50 μg/ml propidium iodide in 0.1% sodium citrate and 0.1% Triton X100 and then analyzed on a Beckman Coulter FC500.

### Apoptosis assays

Programmed cell death was evaluated with the assistance of the DNA fragmentation assay and FACS analysis of propidium iodide (PI), annexin V, and caspase-3 reactivity. For the detection of nucleosomal DNA fragmentation, DNA was extracted using the Puregene kit (Gentra Systems, Minneapolis, MN) and fractionated by electrophoresis on 1.2% agarose gels containing ethidium bromide. For the determination of cells with sub-G0/G1 DNA content, cells were re-suspended in PI/Rnase buffer (BD Pharmingen, San Diego, CA) for 20 min at 37°C in the dark, followed by FACS analysis. Annexin-V reactivity was determined with a phycoerythrin (PE)-labeled antibody (BD Pharmingen) in cells co-stained with 7-AAD (7-amino-actinomycin D). This distinguishes dying cells (annexin^+^AAD^-^) from dead cells (annexin^+^AAD^+^). Activated caspase 3 was determined with a FITC-labeled antibody from BD Pharmingen.

### Gene expression profiling on cDNA macroarrays

The relative mRNA expression of genes involved in regulation of apoptosis, cell cycle progression, NFκB signaling, and cellular stress and toxicity responses was analyzed with GEArray (SuperArray Inc., Bethesda, MD) according to the manufacturer's protocol. Cells were treated for 24 hrs with 0.4 mM and 1 mM CDDO-Im, respectively, followed by preparation of total RNA using TriReagent (Sigma). Five μg from each sample were reverse transcribed into ^32^P-labeled cDNA using MMLV reverse transcriptase (Promega, Madison, WI) and ^32^P-dCTP (NEN, Boston, MA). The resulting cDNA probes were hybridized to gene-specific cDNA fragments spotted in quadruplicates on the GEArray membranes. After stringent washing of the arrays, the signal of the hybridized spots was measured with a STORM PhosphorImager (Molecular Dynamics, Sunnyvale, CA) and normalized to the signal of the housekeeping gene *Gapd*. Array results on six CDDO-Im inducible genes were validated using semi-quantitative RT-PCR.

### Gene microarray hybridization and analysis

cDNA made from total RNA (50 μg) from iMyc^Eμ^-1 and-2 cells was labeled with cyanine 5-conjugated dUTP (Cy5) and cDNA made from pooled mouse cell line RNA (50 μg) was labeled with cyanine 3-conjugated dUTP (Cy3). Microarray hybridizations were performed on Mouse Lymphochip microarrays [53]. After washing, the slides were scanned using an Axon GenePix 4.0 scanner (Axon Instruments Inc., Union City, CA). After normalization, those elements that failed to meet confidence criteria based on signal intensity and spot quality were excluded from analysis. In addition, data were discarded for any gene for which measurements were missing on >30% of the arrays or were not sequence-verified. The Cy5:Cy3 intensity ratios of the remaining spots were log_2 _transformed. To compare normal samples, hierarchical cluster analysis was performed using the Gene Cluster and Treeview programs [54].

### Plasmacytoma induction in iMyc^Eμ ^mice treated with CDDO-Im

Transgenic iMyc^Eμ ^mice on the PCT-susceptible background of BALB/c were fed Purina Mouse Chow (PMI Feeds, St Louis, MO) and acidified water *ad libitum*. All experiments were performed in a conventional barrier-protected colony under NCI Animal Study Protocol LG-028. PCT were induced with a single i.p. injection of 0.2 ml pristane (Aldrich, Milwaukee, WI) on day 1. Beginning on day 7 post-pristane and continuing throughout the observation period of 60 days, primed mice of this sort (n = 25) were treated with three weekly i.p. injections of either 50 μl CDDO-Im solution (2 mg/ml polyethylene glycol 400 [PEG 400]) (n = 14) or 50 μl vehicle control (PEG 400, n = 11).

PCT were diagnosed on days 30 and 60 post pristane by finding 10 or more hyperchromatic, enlarged, aberrant plasma cells in cytofuged preparations of ascites cells. In mice where there were less than 50 tumor cells per slide, a confirmatory smear was obtained.

## Competing interests

The author(s) declare that they have no competing interests.

## Authors' contributions

Seong-Su Han determined Superarray gene expression profiles and Myc protein levels; Liangping Peng and Seung-Tae Chung harvested and transplanted tumors, cultured cells, and performed cytological and FACS analyses; Sungho Maeng validated gene array results using RT-PCR; Art Shaffer determined gene expression profiles on mouse Lymphochips; Wendy DuBois conducted the tumor induction study in mice; Michael Sporn provided CDDO-Im and contributed critical insights; and Siegfried Janz designed the study and wrote and approved the article.

## Supplementary Material

Additional File 1 contains a table of discordantly regulated genes upon treatment with CDDO-Im Click here for file

Additional File 2 contains a table of RT-PCR primers used for gene array validation Click here for file
