# Supplementary material for: CDDO-Imidazolide inhibits growth and survival of c-Myc-induced mouse B cell and plasma cell neoplasms
Source: Mol Cancer. 2006 Jun 7;5:22. doi: 10.1186/1476-4598-5-22 (PMC1553469; doi:10.1186/1476-4598-5-22)
Supplement: Additional File 1 — contains a table of discordantly regulated genes upon treatment with CDDO-Im [file 1476-4598-5-22-S1.pdf]

Additional File 1: Discordantly regulated genes upon treatment with CDDO-Im

Gene symbol	Gene name	Gene function	Cell line		Array[1]	Pos.[2]
			iMyc-1	iMyc-2		
Asc-pending	Apoptosis-associated speck-like protein containing a CARD (pending)	CARD family	-2.3	-	Apo	D1
Bax	Bcl2-associated X protein	Bcl-2 family	-3.0	-	Apo	A2
Birc1a	baculoviral IAP repeat-containing 1a	IAP family	-2.3	-	Apo	D3
Birc1e	baculoviral IAP repeat-containing 1e	IAP family	-2.1	-	Apo	F3
Casp1	caspase 1	caspase family	3.0	-	Tox	F1
Cdc25b	cell division cycle 25 homolog B (S. cerevisiae)	M phase	3.8	-	Cycle	H3
Cyp2b10	cytochrome P450, family 2, subfamily b, polypeptide 10	oxidative stress	3.0	-	Tox	B3
Fadd	Fas (TNFRSF6)-associated via death domain	death domain family	2.4	-	Apo	D7
Gsr	glutathione reductase 1	oxidative stress	2.9	-	Tox	B6
Hsf1	heat shock factor 1	necrosis	2.5	-	Tox	G6
Ikbkb	inhibitor of kappaB kinase beta	signal transduction kinase	12.3	-	NFkB	D3
Ikbkg	inhibitor of kappaB kinase gamma	signal transduction kinase	3.3	-	NFkB	E3
Map2k3	mitogen activated protein kinase kinase kinase 2	signal transduction kinase	3.1	-	NFkB	G5
Myd88	myeloid differentiation primary response gene 88	death domain family	2.2	-	Apo	D7
Rad23a	RAD23a homolog (S. cerevisiae)	necrosis	2.2	-	Tox	B10
Ripk1	receptor (TNFRSF)-interacting serine-threonine kinase 1	death domain family	-2.2	-	Apo	G8

Skp2	S-phase kinase-associated protein 2 (p45)	cell cycle (G1)	2.2	-	Cycle	G11
Tlr4	toll-like receptor 4	transmembrane receptor	2.0	-	NFkB	G10
Tnfrsf8	tumor necrosis factor receptor superfamily, member 8 (CD30)	TNF receptor family	3.4	-	Apo	E10
Tnfrsf10b	tumor necrosis factor receptor superfamily, member 10b (DR5)	TNF receptor family	-2.7	-	Apo	C9
Traf5	Tnf receptor-associated factor 5	Traf family of adaptor proteins	106	-	Apo	E12
Agt	angiotensinogen	NFkB responsive genes	-	2.1	NFkB	A1
Apaf1	apoptotic protease activating factor 1	p53& ATM pathways	-	-2.2	Cycle	B1
Atm	ataxia telangiectasia mutated homolog (human)	p53& ATM pathways	-	5.8	Apo	E1
Bcl2a1d	B-cell leukemia/lymphoma 2 related protein A1d	Bcl-2 family	-	5.8	Apo	D2
Birc4	baculoviral IAP repeat-containing 4	IAP family	-	-4.8	Apo	A4
Birc6	baculoviral IAP repeat-containing 6	IAP family	-	-2.0	Apo	C4
Cflar	CASP8 and FADD-like apoptosis regulator (Cash)	Death effector domain family	-	2.2	Apo	G4
Casp7	caspase 7	caspase family	-	-3.4	Apo	G5
Casp8ap2	caspase 8 associated protein 2	Death effector domain family	-	-2.2	Apo	A6
Ccng2	cyclin G2	cell cycle (S)	-	-3.0	Cycle	C3
Cdc25b	cell division cycle 25 homolog B (S. cerevisiae)	cell cycle (S)	-	-4.8	Cycle	H3
Cdc37	cell division cycle 37 homolog (S. cerevisiae)	cell cycle (G1)	-	-3.0	Cycle	B4
Cdc6	cell division cycle 6 homolog (S. cerevisiae)	cell cycle (S)	-	-4.8	Cycle	D4
Cdk7	cyclin-dependent kinase 7	cell cycle (S)	-	-2.3	Cycle	B5
Chek1	checkpoint kinase 1 homolog (S. pombe)	p53& ATM pathways	-	-3.2	Apo	D6
Cideb	cell death-inducing DNA fragmentation factor, alpha subunit-like effector B	CIDE domain family	-	-3.8	Apo	F6
Crya2	crystallin, alpha B	oxidative stress	-	9.1	Tox	D2

Csf1	colony stimulating factor 1 (macrophage)	NFkB responsive genes	-	3.1	NFkB	F1
Cyp1a1	cytochrome P450, family 1, subfamily a, polypeptide 1	oxidative stress	-	3.6	Tox	F2
Cyp1a2	cytochrome P450, family 1, subfamily a, polypeptide 2	oxidative stress	-	7.8	Tox	G2
Cyp3a11	cytochrome P450, family 3, subfamily a, polypeptide 11	oxidative stress	-	11.9	Tox	E3
Cyp4a14	cytochrome P450, family 4, subfamily a, polypeptide 14	oxidative stress	-	6.9	Tox	G3
Ddit3	DNA-damage inducible transcript 3	growth and senescence	-	2.1	Tox	B4
Dffa	DNA fragmentation factor, alpha subunit	CIDE domain family	-	2.7	Apo	A7
E2f4	E2F transcription factor 4	cell cycle (G1)	-	-2.7	Cycle	G7
Fmo1	flavin containing monooxygenase 1	necrosis	-	5.0	Tox	C5
Gpx2	glutathione peroxidase 2	oxidative stress	-	15.6	Tox	A6
Il1a	interleukin 1 alpha	inflammation	-	31.3	Tox	D8
Il12b	interleukin 12b	NFkB responsive genes	-	55.5	NFkB	G3
Il1rak	interleukin-1 receptor-associated kinase 1	adaptor protein	-	-4.5	NFkB	D4
Il6	interleukin 6	inflammation	-	13.2	Tox	F8
Map3k2	mitogen activated protein kinase kinase kinase 2	signal transduction kinase	-	-2.3	NFkB	C6
Mapk14	mitogen activated protein kinase 14	signal transduction kinase	-	-3.8	NFkB	F6
Max	Max protein	transcription factor	-	-2.0	NFkB	B7
Mcmd2	minichromosome maintenance deficient 2 mitotin (S. cerevisiae)	cell cycle (S)	-	-4.8	Cycle	F8
Mcmd5	minichromosome maintenance deficient 5 mitotin (S. cerevisiae)	cell cycle (S)	-	-2.9	Cycle	A9
Mcmd6	minichromosome maintenance deficient 6 mitotin (S. cerevisiae)	cell cycle (S)	-	-3.2	Cycle	B9
Mcmd7	minichromosome maintenance deficient 7 mitotin (S. cerevisiae)	cell cycle (S)	-	-2.2	Cycle	C9
Mre11a	meiotic recombination 11 homolog A (S. cerevisiae)	p53& ATM pathways	-	-2.2	Cycle	F9

Myc	myelocytomatosis oncogene	transcription factor	-	-3.3	Cycle	C7
Nbn	nibrin	p53& ATM pathways	-	-2.0	Cycle	G9
Nos2	nitric oxide synthase 2, inducible, macrophage	inflammation	-	11.6	Tox	F9
Ptgs2	prostaglandin-endoperoxide synthase 2	oxidative stress	-	6.6	Tox	A10
Rad53	CHK2 checkpoint homolog (S. pombe)	p53& ATM pathways	-	-3.2	Cycle	G10
Raf1	v-raf-1 leukemia viral oncogene 1	transcription factor	-	-2.4	NFkB	G8
Rbl1	retinoblastoma-like 1 (p107)	cell cycle (G1)	-	-2.5	Cycle	B11
Scya21a	chemokine (C-C motif) ligand 21a (leucine)	inflammation	-	2.5	Tox	E10
Scya21b	chemokine (C-C motif) ligand 21b (leucine)	inflammation	-	36.7	Tox	F10
Sod2	superoxide dismutase 2, mitochondrial	oxidative stress	-	-2.9	Tox	E11
Timp3	tissue inhibitor of metalloproteinase 3	p53& ATM pathways	-	2.8	Cycle	A12
Tnfrsf9	tumor necrosis factor receptor superfamily, member 9	TNF receptor family	-	3.0	Apo	F10
Tnfsf10	tumor necrosis factor (ligand) superfamily, member 10	apoptosis signaling	-	-11.1	Tox	H11
Tnfsf13	tumor necrosis factor (ligand) superfamily, member 13 (April)	TNF receptor family	-	61.2	Apo	B1
Traf1	Tnf receptor-associated factor 1	Traf family of adaptor proteins	-	3.4	NFkB	B12
Traf4	Tnf receptor-associated factor 4	Traf family of adaptor proteins	-	2.1	NFkB	E12
Traf6	Tnf receptor-associated factor 6	Traf family of adaptor proteins	-	-2.7	Apo	F12
Traip	TRAF-interacting protein	Traf family of adaptor proteins	-	2.7	Apo	G12
Trp53	transformation related protein 53	p53& ATM pathways	-	-4.2	Cycle	B12
Vcam1	vascular cell adhesion molecule 1	NFkB responsive genes	-	7.6	NFkB	H12
Xrcc1	X-ray repair complementing defective repair in Chinese hamster cells 1	DNArepair	-	-3.4	Tox	E12

[1] GEArray Q series mouse cDNA gene arrays (SuperArray Bioscience Corporation, Gaithersburg, MD) included the MM-001 cell cycle array (Cycle), MM-002 apoptosis array (Apo), MM-012 stress and toxicity array (Tox) and MM-016 NFkB signaling array (NFkB).

[2] Array position
