# Supplementary material for: CDDO-Imidazolide inhibits growth and survival of c-Myc-induced mouse B cell and plasma cell neoplasms
Source: Mol Cancer. 2006 Jun 7;5:22. doi: 10.1186/1476-4598-5-22 (PMC1553469; doi:10.1186/1476-4598-5-22)
Supplement: Additional File 2 — contains a table of RT-PCR primers used for gene array validation [file 1476-4598-5-22-S2.pdf]

Additional File 2: RT-PCR primers used for gene array validation

Gene symbol	Forward primer	Backward primer
Fmo4	GAAGCCCAGCATTCCACTTTTG	TGTTGAGAGCAGCACCAATCTG
Casp14	TAACACCACCTTCCTTTCGCTG	ATGGCATCTTCTTCCTTGGGAC
Hmox1	AAAGACCAGAGTCCCTCACAGATGG	TTCCCACCCACCCCTCAAAAGATAG
Cyp2a4	TTCCTCAGCGTCCTGGTTTTGATG	AATGAAAGCACCGTTCGTCTTCCG
Cyp2b9	TGTGCTCTCTCTCTTCTTTGCTGG	TGCTCAGGATTGAACTTGTCTGG
Cyp2c29	GGAAGAACTGAGGAAAACCAAAGGC	TGTTGTGCTTGTTGTCTCTGTCCC
